# Enhancement of radiosensitization by metal-based nanoparticles in cancer radiation therapy

**DOI:** 10.7497/j.issn.2095-3941.2014.02.003

**Published:** 2014-06

**Authors:** Xiang-Yu Su, Pei-Dang Liu, Hao Wu, Ning Gu

**Affiliations:** ^1^Department of Oncology, Zhongda Hospital of Southeast University, Nanjing 210009, China; ^2^Jiangsu Key Laboratory for Biomaterials and Devices, Southeast University, Nanjing 210009, China

**Keywords:** Nanoparticles, radiation tolerance, cancer, radiotherapy

## Abstract

Radiation therapy performs an important function in cancer treatment. However, resistance of tumor cells to radiation therapy still remains a serious concern, so the study of radiosensitizers has emerged as a persistent hotspot in radiation oncology. Along with the rapid advancement of nanotechnology in recent years, the potential value of nanoparticles as novel radiosensitizers has been discovered. This review summarizes the latest experimental findings both *in vitro* and *in vivo* and attempts to highlight the underlying mechanisms of response in nanoparticle radiosensitization.

## Introduction

Cancer is one of the leading causes of mortality among humans, with more than 760 million deaths every year^1^. Although radiation therapy plays an important role in cancer treatment, the resistance of tumor cells to radiation therapy still remains a serious concern. Therefore, the study of radiosensitizers has emerged as a persistent hotspot in radiation oncology^2^. Nanotechnology has provided new and powerful tools for imaging, diagnosing, and treating cancer^3^. In cancer radiotherapy, the concept of high-Z material radiation dose enhancement has been known for several decades^4^. Nanoparticles, especially noble metal nanoparticles, may be useful in enhancing the efficacy of radiotherapy because of their unique physical and chemical properties^5^. To date, several different nanoparticles have been applied as potential tumor-selective radiosensitizers. In this study, we will focus on the *in vitro* and *in vivo* experimental findings as well as the underlying mechanisms of response in metal-based nanoparticle radiosensitization.

## Gold nanoparticles (GNPs) and radiosensitization

In recent years, GNPs have been widely used and analyzed in radiation therapy because of their extremely small size, good biocompatibility, and ease in chemical modification. The number of reports on GNP radiosensitization has rapidly increased^6^.

### Monte Carlo (MC) calculations

MC calculations facilitate the accurate estimation of the dose enhancement effect caused by GNPs^7^. In a preliminary study, Cho^8^ evaluated the dose enhancement effect of GNPs with both kilovoltage (Ir-192 and 140 kV) and megavoltage (4 and 6 MV) photons. The dose enhancement over the tumor volume considered for the 140 kVp X-ray case can be at least a factor of 2 at an achievable gold (Au) concentration of 7 mg Au/g tumor, assuming that no Au is outside the tumor. The tumor dose enhancement for cases involving the 4 and 6 MV photon beams based on the same assumption ranged from approximately 1% to 7%, depending on the amount of Au within the tumor and the photon beam qualities. For the Ir-192 case, the dose enhancement ratio of 5% to 31% depending on the radial distance and Au concentration has been reported. In another study by Cho^9^, the feasibility of GNPs interacting with low energy photons was investigated using MC calculations. Brachytherapy sources of I-125, 50 kVp X-rays, and Yb-169 were used to calculate the macroscopic dose enhancement factor (MDEF), which is a ratio of the average dose in the tumor region with and without the presence of GNPs during tumor irradiation. For a tumor loaded with 18 mg Au/g, respective MDEFs of 116%, 92%, and 108% were reported for I-125, 50 kVp, and Yb-169, respectively, at the distance of 1.0 cm from the center of the source. However, at 7 mg Au/g, the corresponding MDEFs decreased to 68%, 57%, and 44%. These data suggest that even at low-energy radiation brachytherapy, GNPs could also serve as radiation sensitizers.

Limited studies have shown that MC calculations contribute little benefit at the MV energies. Lechtman^10^ used MC calculations to explore the effects of different irradiation energies to define the optimal clinical use of GNPs. Photon sources included brachytherapy seeds Pd-103 and I-125, high dose rate sources Yb-169 and Ir-192, and external beam sources 300 kVp and 6 MV. The results indicated that doubling of the prescribed dose in a tumor can be achieved if the amount of GNPs required is approximately 300 times greater for the 6 MV source compared with lower energy brachytherapy sources. Thus, therapeutic uses of GNPs in radiosensitization with a 6 MV photon source may be not clinically feasible.

Most studies have attributed GNP radiosensitization to increased photoelectric photon absorption by high-Z materials at kilovoltage photon energies. However, if sensitization occurs by this physical mechanism, effects would not be predicted to occur at clinically relevant megavoltage energies dominated by Compton interactions^11^. From the mechanism’s point of view, GNPs will create additional short-range secondary electrons once activated by high-energy electron beams. Therefore, the enhancement of radiosensitivity is due to the production of these low-energy electrons caused by the increased absorption of ionizing radiation energy by the particles^12^.

### *In vitro* studies

*In vitro* radiation enhancement of GNPs, which can be simulated and calculated by the MC method, has been extensively examined.

GNP size may be an important factor in increasing the radiation cytotoxicity. Chithrani^13^ investigated the impact of GNP size, concentration, and radiation energy on *in vitro* radiosensitization in HeLa cells and found that 50 nm GNPs had better radiosensitizing ability than 14 and 74 nm GNPs with 220 kVp X-rays (DEFs were 1.43, 1.2, and 1.25, respectively). The magnitude of radiosensitization was found to be dependent on GNP concentration with 50 nm GNPs and correlated with the number of intracellular nanoparticles, but not on the total amount of intracellular Au when different-sized nanoparticles were considered.

Surface properties may also influence the radiosensitization of GNPs. Kong *et al*.^14^ reported that the localized uptake and binding of GNPs at selected locations in cancer cells could be achieved by modifying the surface properties of GNPs. They developed two types of GNPs, Glu-GNPs and AET-GNPs, to enhance localized uptake and binding to the cancer cell. Different radiations, such as 200 kVp X-rays and gamma rays, were applied to radiation therapy of the cells, with naked GNPs or functional GNPs. The results showed that radiotherapy in association with functional GNPs killed significantly more breast cancer cells compared with the naked GNPs. In this study, the naked GNPs were neutral and passively bound to cells. However, the biomolecule-modified GNPs could selectively target locations at the subcellular level. The active and specific binding significantly increased the local concentration of functional GNPs, and subsequently enhanced the cytotoxicity of radiation. Liu *et al*.^15^ also confirmed that PEG-GNPs could enhance the cell radiation therapeutic sensitivity in murine breast cancer EMT-6 cells and colon carcinoma CT26 cells. The percentage of surviving cells after irradiation decreased by approximately 2% to 45%. Zhang *et al*. recently examined HeLa cancer cells using GSH-GNPs or BSA-GNPs irradiated under gamma-rays from ^137^Cs (photon energy 662 keV). The sensitization enhancement ratio (SER) of GSH-GNPs was 1.30, which was higher than that of BSA-GNPs (1.21) for all radiation doses. The radiation enhancement effects of GSH- and BSA-GNPs may have been caused by the enhanced DNA damage induced by the photoelectric effect and Compton scattering of the heavy metal. In addition, GSH-GNPs showed stronger radiation enhancement than BSA-GNPs, which could be attributed to the improved cell uptake of the hydrodynamically smaller GSH-GNPs (2.4 nm) relative to that of the BSA-GNPs (6 nm)^16^.

Radiosensitization was lower at megavoltage energies but was considerably greater than those predicted by MC simulations. McMahon *et al*.^17^ demonstrated that GNPs also had radiosensitization effect on MDA-MB-231 cells at 6 or 15 MV X-ray energies. Clonogenic cell survival assay was used to determine the enhancement of radiosensitivity by GNPs. The SERs were found to be 1.24 and 1.18 for 6 and 15 MV irradiations, respectively. Previous studies have confirmed that greater uptake of GNPs by cells may induce increased radiation effect. A recent study^18^ has shown that Glu-GNPs combined with radiation induced significant growth inhibition in triple-negative breast cancer cell lines (MDA-MB-231) compared with radiation alone at 6 MV X-ray, and 49 nm Glu-GNPs induced stronger radiosensitivity than 16 nm Glu-GNPs with corresponding SERs of 1.86 and 1.49. Notably, GNP sensitization may be cell-specific. In MDA-MB-231 cells, SERs of 1.41, 1.29, and 1.16 were achieved using 160 kVp, 6 MV, and 15 MV X-ray energies, respectively. However, no significant effect was observed in DU145 human prostate cancer cells in the presence of kV or MV energies (SER: 0.97 to 1.08), despite GNPs uptake occurring in these cell lines^19^. Several studies have confirmed that GNPs radiosensitization is substantially greater than MC simulation at megavoltage energies. However, the radiation damage to the cells lining the vasculature should be considered in the complex structure of tumors involving microvasculature. Rahman *et al*.^20^ recently evaluated endothelial dose enhancement factor (EDEF) to bovine aortic endothelial cells (BAECs) using 80 and 150 kV X-ray energies as well as 6 and 12 MeV electrons. The EDEF is defined to be the ratio of the dose given to the control cell culture (i.e., without GNPs) that produces 90% survival divided by the dose given to the cells treated with GNPs that produces 90% survival. They observed EDEFs of 24.6, 2.2, 4.0, and 4.1 for 80 kV X-rays, 150 kV X-rays, 6 MeV electrons, and 12 MeV electrons at 1 mM GNPs concentration, respectively. The results clearly showed that GNPs effectively enhanced the radiation effects on BAECs in conjunction with irradiation by kilo-voltage-energy-range X-ray beams. Slightly less radiation dose enhancement was observed when using high-energy (megavoltage range) electron beams.

### *In vivo* studies

In the pioneering vivo study of Hainfeld *et al*.^21^, in combination with 250 kVp X-ray, the 1.9 nm GNPs were injected intravenously into mammary tumor-bearing mice. Results showed 86% one-year survival using the new method compared with the 20% for X-rays alone. In the following study, they also found that GNPs still had significant radiosensitization on tumor resistant cells^22^. Interestingly, significant *in vivo* tumor growth delay and increased survival were observed in a mouse model with B16F10 murine melanoma^23^ despite the low radiosensitization effect of citrate-coated GNPs found *in vitro* clonogenic assays, with GNPs achieving DEFs of 1.08. The median survival time was 20 d for non-irradiated mice, 55 d for 6 MV radiation only, and 65 days for GNP radiation groups. The authors implied that using multiple fractions may induce more apoptotic cells and thereby improve the therapeutic ratio and survival of tumor-bearing mice in the combination therapy of GNPs and radiation. Previous *in vitro* studies have shown that 50 nm GNPs had stronger radiation sensitivity than 14 and 74 nm GNPs. However, *in vivo* radiosensitization studies of 4.8, 12.1, 27.3, and 46.6 nm PEG-coated GNPs showed that 12.1 nm GNPs had the strongest sensitization effects^24^. More recently, intravenously injected GNPs for X-ray imaging and radiotherapy enhancement of intracerebral malignant gliomas were tested. Mice treated with GNPs and radiation (30 Gy) demonstrated 50% long-term (>1 year) tumor-free survival, whereas all mice treated with radiation only died^25^.

### Mechanism of GNP radiosensitization

The underlying mechanisms of GNPs as radiosensitizers in cancer radiotherapy have been reported recently. The role of double strand breaks (DSB) to the DNA is critical to the response of cancer cells to radiation^26^. Previous studies have shown that γ-H2AX expression is a sensitive DSB indicator. Ngwa *et al*.^27^ observed that for the 28 keV beam (I-125 brachytherapy seeds), the average number of γ-H2AX foci per cell was evidently higher for HeLa cells treated by GNPs plus radiation. For higher energies, Xu *et al*.^28^ successfully prepared Au nanorods and utilized them for radiation with 6 MV X-ray in A375 melanoma cells. The addition of GNPs enhanced the radiosensitivity of A375 cells with a SER of 1.14 and increased more radiation-induced DSBs by γ-H2AX expression. By contrast, no evidence of GNPs increasing radiation-induced DSBs was found by Jain *et al*.^19^. Ionizing radiation is known to generate reactive oxygen species (ROS), such as HO•, O_2_• ^–^, and H_2_O_2_, through the radiolysis of H_2_O molecules. These ROS have a strong destructive effect on the DNA because of their unpaired electron^29^. Moreover, Geng *et al*.^30^ showed that Glu-GNPs enhanced the production of intracellular ROS when irradiated with 90 kVp or 6 MV X-rays in SKOV-3 human ovarian cancer cells. These results indicate that increased ROS formation when radiation interacts with GNPs may be one of the mechanisms that mediate GNP radiosensitization. Another possible mechanism for GNP-mediated radiosensitization is that GNPs induce cell apoptosis and regulate cell cycle. Xu *et al*.^28^ found that irradiation with GNPs on A375 melanoma cells induced a range of cell line-specific responses, including decreased clonogenic survival and increased apoptosis. Roa *et al*.^31^ observed that GNPs accelerated the G_1_/S phase of the cell cycle and arrested DU-145 cancer cells in the G_2_/M phase, confirming how GNPs affect the regulation of the cell cycle to sensitize DU-145 cancer cells to radiotherapy. Furthermore, Roa *et al*.^31^ explored the mechanism of Glu-GNPs mediated cell cycle changes and found that G_2_/M arrest was accompanied by the downregulation of p53 and cyclin A expression and the upregulation of cyclin B1 and cyclin E.

## Silver nanoparticles (AgNPs) and radiosensitization

Accumulated studies have confirmed that AgNPs, an integral component of metal nanomaterials, had obvious anti-tumor capabilities *in vitro*. Given the similar physicochemical properties with GNPs, AgNPs have attracted much interest in radiotherapy.

### *In vitro* studies

Xu *et al*.^32^ tested the radiosensitization effects of AgNPs with different sizes (20, 50, and 100 nm) in glioma cells (rat C6 glioma cells, human U251 and SHG-44 glioma cells) and demonstrated that AgNPs could function to enhance radiation-induced necrosis of glioma cells. They found that both 20 and 50 nm AgNPs significantly enhanced radiation sensitivity of U251 cells, with 20 nm particles performing better than 50 nm ones, while the effect of 100 nm AgNPs was significantly weaker. A similar particle size-dependent radiation sensitization effect was also observed for C6 and SHG-44 cells. Thenceforth, several similar studies proved that AgNPs had radiation sensitization effect on MGC803 gastric cells^33^, U231 breast cancer cells^34^, and A549 lung cancer cells^35^.

### *In vivo* studies

Whether the *in vitro* findings could be applied *in vivo* remain unclear because of the disparity of microenvironments and condition controllabilities. Liu *et al*.^36^ treated C6 glioma-bearing rats with a single dose of 10 Gy using 6 MV X-rays radiation alone or in combination with intratumoral administration of AgNPs. The mean survival times were 100.5 and 98 d, the corresponding percent increase in life spans were 513.2% and 497.7%, and the cure rates were 41.7% and 38.5% at 200 d for the 10 or 20 μg AgNPs and radiation combination groups, respectively. By contrast, the mean survival times for irradiated controls, 10 and 20 μg AgNPs alone and untreated controls were 24.5, 16.1, 19.4, and 16.4 d, respectively. Finally, the results showed the therapeutic efficacy of AgNPs in combination with radiotherapy without apparent systemic toxicity, thus suggesting the clinical potential of AgNPs in improving the outcome of malignant glioma radiotherapy. Therefore, the findings from this study will be critical for successful translation of this approach in clinic.

### Mechanism of AgNPs radiosensitization

The anti-tumor capability of AgNPs has been primarily attributed to inducing apoptosis, activating the oxidative stress, and influencing membrane fluidity^37^^-^^39^. Currently, the mechanisms of AgNPs radiosensitization are not fully determined. Therefore, several studies have proposed that the mechanism of radiosensitization by AgNPs may be related to the release of Ag^+^ cation from the Ag nanostructures inside cells. Ag^+^ cation has the ability to capture electron and thus functions as an oxidative agent, which could further reduce the ATP content of the cell and increase production of ROS. Liu *et al*. found that when gliomas were treated with AgNPs followed by radiotherapy, a cooperative antiproliferative and proapoptotic effect was obtained^36^.

## Other metal nanomaterials and radiosensitization

Using GNPs and AgNPs as radiosensitizers have been confirmed by several experimental and MC simulation studies. However, scientists showed great interest in radiosensitization caused by other nanomaterials. Germanium (Ge) is a naturally occurring metalloid with semiconductor properties^40^. Lin *et al*.^41^ demonstrated that nanoGe can enhance the radiosensitivity of Chinese hamster ovary K1 cells. They found that nanoGe caused a higher level of DNA damage by the comet assay and caused cell cycle arrest at G_2_/M phase. Given that the atomic number of platinum is similar to that of Au, Porcel *et al*.^42^ proposed a new strategy based on the combination of platinum nanoparticles with irradiation by fast ions effectively used in hadron therapy. They observed that platinum nanoparticles strongly enhanced lethal damage in DNA, with an efficiency factor close to 2 for DSB. The authors supposed that the enhanced sensitization of platinum nanoparticles was due to reinforcing the energy deposition in the close vicinity of the metal. Considered as one of the least toxic heavy metals, bismuth has been widely used in industry as well as in biological and medical sciences^43^. Recently, bismuth nanoparticles have drawn great attention for application in biological sciences such as bioimaging, biosensing, biomolecular detection, and X-ray radiosensitizing. Hossain *et al*.^44^ found that bismuth nanoparticles showed higher dose enhancements than Au and platinum nanoparticles for a given nanoparticle size, concentration, and location. At 350 mg/g, bismuth nanoparticles provided 1.25 and 1.29 times higher dose enhancements than GNPs and platinum nanoparticles respectively when irradiated by a 50 kVp source. Correspondingly, Auger electrons from bismuth nanoparticles provided 2 to 2.4 times higher enhancement than the other two kinds of nanoparticles. Hence, the highest DEFs may be achieved for nanoparticles located closest to the nucleus, where energy depositions from short range Auger electrons were the maximum.

## Conclusion

With the rapid development of nanotechnology in the biomedical field, nanomaterials have been widely used in the diagnosis and treatment for disease. Numerous pre-clinical studies *in vitro* and *in vivo* have proved the potential value of metal-based nanomaterials as radiosensitizers in cancer treatment. Various studies have indicated that radiosensitizing ability could be influenced by nanomaterial size, concentration, surface coating, and the radiation energy. Further systematic and comparative studies are needed to achieve the best *in vivo* radiosensitization effect. In addition, although the exact molecular mechanisms of radiosensitization are elusive, the role of autophagy (programmed cell death type II) in this effect should be considered. Finally, several other issues, such as nanomaterial metabolism *in vivo*, biodistribution, and cumulative toxicity (biosecurity) *in vivo* still remain unaddressed. In the future, we believe that all these problems will eventually be resolved by the development of nanomedicinal technology.
